# Cutaneous Infrared Thermometry for Detecting Febrile Patients 

**DOI:** 10.3201/eid1408.080059

**Published:** 2008-08

**Authors:** Pierre Hausfater, Yan Zhao, Stéphanie Defrenne, Pascale Bonnet, Bruno Riou

**Affiliations:** * UPMC Univ Paris 06, Paris, France; † Centre Hospitalier Universitaire Pitié-Salpêtrière, Paris; ‡ Zhongnan Hospital, Wuhan, People’s Republic of China

**Keywords:** Fever, mass detection, cutaneous infrared thermometry, infectious diseases, emergency, dispatch

## Abstract

We assessed the accuracy of cutaneous infrared thermometry, which measures temperature on the forehead, for detecting patients with fever in patients admitted to an emergency department. Although negative predictive value was excellent (0.99), positive predictive value was low (0.10). Therefore, we question mass detection of febrile patients by using this method.

Recent efforts to control spread of epidemic infectious diseases have prompted health officials to develop rapid screening processes to detect febrile patients. Such screening may take place at hospital entry, mainly in the emergency department, or at airports to detect travelers with increased body temperatures (*1*–*3*). Infrared thermal imaging devices have been proposed as a noncontact and noninvasive method for detecting fever (*4*–*6*). However, few studies have assessed their capacity for accurate detection of febrile patients in clinical settings. Therefore, we undertook a prospective study in an emergency department to assess diagnostic accuracy of infrared thermal imaging.

## The Study

The study was performed in an emergency department of a large academic hospital (1,800 beds) and was reviewed and approved by our institutional review board (Comité de Protection des Personnes se Prêtant à la Recherche Biomédicale Pitié-Salpêtrière, Paris, France). Patients admitted to the emergency department were assessed by a trained triage nurse, and several variables were routinely measured, including tympanic temperature by using an infrared tympanic thermometer (Pro 4000; Welch Allyn, Skaneateles Falls, NY, USA), systolic and diastolic arterial blood pressure, and heart rate.

**Figure 1:**
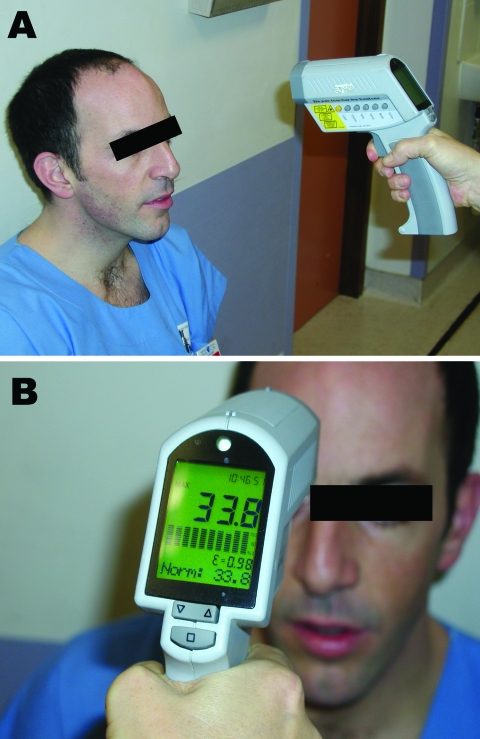
Measurement of cutaneous temperature with an infrared thermometer. A) The device is placed 20 cm from the forehead. B) As soon as the examiner pulls the trigger, the temperature measured is shown on the display. Used with permission.

Tympanic temperature was measured twice (once in the left ear and once in the right ear). This temperature was used as a reference because it is routinely used in our emergency department and is an appropriate estimate of central core temperature (*7*–*9*). Cutaneous temperature was measured on the forehead by using an infrared thermometer (Raynger MX; Raytek, Berlin, Germany) (Figure 1). Rationale for an infrared thermometer device instead of a larger thermal scanner was that we wanted to test a method (i.e., measurement of forehead cutaneous temperature by using a simple infrared thermometer) and not a specific device. The forehead region was chosen because it is more reliable than the region behind the eyes (*5*,*10*). The latter region may not be appropriate for mass screening because one cannot accurately measure temperature through eyeglasses, which are worn by many persons. Outdoor and indoor temperatures were also recorded.

The main objective of our study was to assess diagnostic accuracy of infrared thermometry for detecting patients with fever, defined as a tympanic temperature >38.0°C. The second objective was to compare measurements of cutaneous temperature and tympanic temperature, with the latter being used as a reference point. Data are expressed as mean ± standard deviation (SD) or percentages and their 95% confidence intervals (CIs). Comparison of 2 means was performed by using the Student *t* test, and comparison of 2 proportions was performed by using the Fisher exact method. Bias, precision (in absolute values and percentages), and number of outliers (defined as a difference >1°C) were also recorded. Correlation between 2 variables was assessed by using the least square method. The Bland and Altman method was used to compare 2 sets of measurements, and the limit of agreement was defined as ± 2 SDs of the differences (*11*). We determined the receiver operating characteristic (ROC) curves and calculated the area under the ROC curve and its 95% CI. The ROC curve was used to determine the best threshold for the definition of hyperthermia for cutaneous temperature to predict a tympanic temperature >38°C. We performed multivariate regression analysis to assess variables associated with the difference between tympanic and infrared measurements. All statistical tests were 2-sided, and a p value <0.05 was required to reject the null hypothesis. Statistical analysis was performed by using Number Cruncher Statistical Systems 2001 software (Statistical Solutions Ltd., Cork, Ireland).

A total of 2,026 patients were enrolled in the study: 1,146 (57%) men and 880 (43%) women 46 ± 19 years of age (range 6–103 years); 219 (11%) were >75 years of age, and 62 (3%) had a tympanic temperature >38°C. Mean tympanic temperature was 36.7°C ± 0.6°C (range 33.7°C–40.2°C), and mean cutaneous temperature was 36.7°C ± 1.7°C (range 32.0°C–42.6°C). Mean systolic arterial blood pressure was 130 ± 19 mm Hg, mean diastolic blood pressure was 79 ± 13 mm Hg, and mean heart rate was 86 ± 17 beats/min. Mean indoor temperature was 24.8°C ± 1.1°C (range 20°C–28°C), and mean outdoor temperature was 10.8°C ± 6.8°C (range 0°C–32°C). Reproducibility of infrared measurements was assessed in 256 patients. Bias was 0.04°C ± 0.35°C, precision was 0.22°C ± 0.27°C (i.e., 0.6 ± 0.7%), and percentage of outliers >1°C was 2.3%.

**Figure 2:**
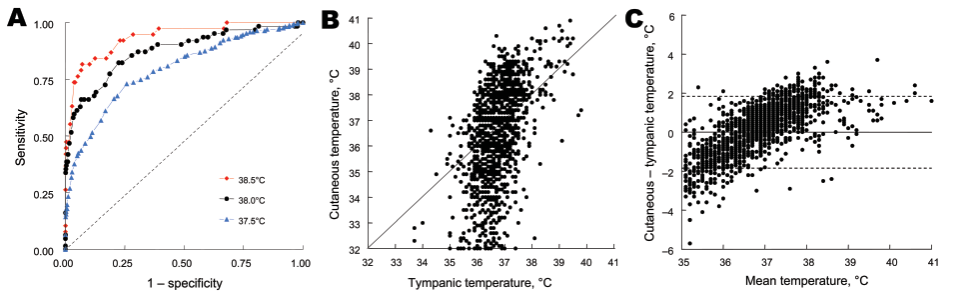
A) Comparison of receiver operating characteristic (ROC) curves showing relationship between sensitivity (true positive) and 1 – specificity (true negative) in determining value of cutaneous temperature for predicting various thresholds of hyperthermia definitions (37.5°C, 38.0°C, and 38.5°C) of tympanic temperature. Areas under ROC curves (95% confidence interval) were 0.935 (0.876–0.966), 0.873 (0.807–0.917), and 0.792 (0.749–0.829), respectively, and all were significantly (p<0.001) different from the identity line (dashed diagonal line). B) Correlation and C) Bland and Altman diagrams comparing cutaneous and tympanic temperature measurements (n = 2,026 patients). Values on the y-axis in panel C represent differences between cutaneous and tympanic temperatures. The solid horizontal line in panel C represents the null difference between cutaneous and tympanic temperatures, and the 2 dashed horizontal lines represent ± 2 standard deviations.

Diagnostic performance of cutaneous temperature measurement is shown in Table 1. For the threshold of the definition of tympanic hyperthermia definition used (37.5°C, 38°C, or 38.5°C), sensitivity of cutaneous temperature was lower than that expected and positive predictive value was low. We attempted to determine the best threshold (definition of hyperthermia) by using cutaneous temperature to predict a tympanic temperature >38°C (Figure 2, panel A). Area under the ROC curve was 0.873 (95% CI 0.807–0.917, p<0.001). The best threshold for cutaneous hyperthermia definition was 38.0°C, a condition already assessed in Table 1. Figure 2, panels B and C shows the correlation between cutaneous and tympanic temperature measurements (Bland and Altman diagrams). Correlation between cutaneous and tympanic measurements was poor, and the infrared thermometer underestimated body temperature at low values and overestimated it at high values. Multiple regression analysis showed that 3 variables (tympanic temperature, outdoor temperature, and age) were significantly (p<0.001) and independently correlated with the magnitude of the difference between cutaneous and tympanic measurements (Table 2).

**Table 1:** Assessment of diagnostic performance of cutaneous temperature in predicting increased tympanic temperature*

Characteristic	Predicted tympanic temperature, °C†		
	>37.5	>38.0	>38.5
Cutaneous temperature threshold, °C‡	37.5	38.0	38.5
Sensitivity	0.76 (0.69–0.82)	0.82 (0.71–0.90)	0.82 (0.67–0.91)
Specificity	0.65 (0.63–0.67)	0.77 (0.76–0.79)	0.90 (0.88–0.91)
Positive predictive value	0.16 (0.14–0.19)	0.10 (0.08–0.13)	0.13 (0.09–0.18)
Negative predictive value	0.97 (0.96–0.98)	0.99 (0.99–1.00)	1.00 (0.99–1.00)
Accuracy	0.66 (0.64–0.68)	0.78 (0.76–0.79)	0.90 (0.89–0.91)

*Values in parenthesis are 95% confidence intervals. †Definition of hyperthermia. ‡Corresponds to the best threshold for a definition of cutaneous hyperthermia determined by using receiver operating characteristic curve.

**Table 2:** Variables correlated with magnitude of the difference between cutaneous and tympanic temperature measurements*

Variables	Coefficient of regression (95% confidence interval)
Tympanic temperature, °C	0.27 (0.17 to 0.37)
Age, y	−0.012 (–0.015 to –0.009)
Outdoor temperature, °C	0.04 (0.03 to 0.05)

*p<0.001 for all comparisons.

## Conclusions

Infrared thermometry does not reliably detect febrile patients because its sensitivity was lower than that expected and the positive predictive value was low, which indicated a high proportion of false-positive results. Ng et al. (*5*) studied 502 patients, concluded that an infrared thermal imager can appropriately identify febrile patients, and reported a high area under the ROC curve value (0.972), which is similar to the area we found in the present study (0.925). However, such global assessment is of limited value because of low incidence of fever in the population. Rather than looking at positive predictive value or accuracy, one should determine negative predictive value. This determination might be of greater consequence if one considers an air traveler population or a population entering a hospital.

Ng et al. (*5*) identified outdoor temperature as a confounding variable in cutaneous temperature measurement. Our study identified age as a variable that interferes with cutaneous measurement, but the role of gender is less obvious. Older persons showed impaired defense (stability) of core temperatures during cold and heat stresses, and their cutaneous vascular reactivity was reduced (*12*,*13*).

Use of a simple infrared thermometry, rather than sophisticated imaging, should not be considered a limitation because this method concerns the relationship between cutaneous and central core temperatures. We can extrapolate our results to any devices that estimate cutaneous temperature and the software used to average it. Our study attempted to detect febrile patients, not infected patients. For mass detection of infection, focusing on fever means that nonfebrile patients are not detected. This last point is useful because fever is not a constant phenomenon during an infectious disease, antipyretic drugs may have been taken by patients, and a hypothermic rather than hyperthermic reaction may occur during an infectious process.

In conclusion, we observed that cutaneous temperature measurement by using infrared thermometry does not provide a reliable basis for screening outpatients who are febrile because the gradient between cutaneous and core temperatures is markedly influenced by patient’s age and environmental characteristics. Mass detection of febrile patients by using this technique cannot be envisaged without accepting a high rate of false-positive results.
